# Genomic Effect of DNA Methylation on Gene Expression in Colorectal Cancer

**DOI:** 10.3390/biology11101388

**Published:** 2022-09-23

**Authors:** Juyeon Hong, Je-Keun Rhee

**Affiliations:** Department of Bioinformatics & Life Science, Soongsil University, Seoul 06978, Korea

**Keywords:** colorectal cancer, DNA methylation, gene expression, regression, ElasticNet, survival analysis, immune system

## Abstract

**Simple Summary:**

Abnormal DNA methylation is known to regulate gene expression, and its features have been frequently observed in colorectal cancer (CRC) patients. In addition, alterations in DNA methylation can be proposed as biomarkers for cancer prognosis, as they occur in the early stage of carcinogenesis. Although numerous studies have attempted to shed light on the impacts of DNA methylation on gene expression, it is still unclear which specific regions regulate gene expression and how they are associated with patient survival. In this study, we elucidated the intricate relationship between DNA methylation and gene expression. Furthermore, we found genes that were influenced by DNA methylation and were associated with survival; these genes were mainly enriched in immune-related pathways.

**Abstract:**

The aberrant expression of cancer-related genes can lead to colorectal cancer (CRC) carcinogenesis, and DNA methylation is one of the causes of abnormal expression. Although many studies have been conducted to reveal how DNA methylation affects transcription regulation, the ways in which it modulates gene expression and the regions that significantly affect DNA methylation-mediated gene regulation remain unclear. In this study, we investigated how DNA methylation in specific genomic areas can influence gene expression. Several regression models were constructed for gene expression prediction based on DNA methylation. Among these models, ElasticNet, which had the best performance, was chosen for further analysis. DNA methylation near transcription start sites (TSS), especially from 2 kb upstream to 7 kb downstream of TSS, had an essential regulatory role in gene expression. Moreover, methylation-affected and survival-associated genes were compiled and found to be mainly enriched in immune-related pathways. This study investigated genomic regions in which methylation changes can affect gene expression. In addition, this study proposed that aberrantly expressed genes due to DNA methylation can lead to CRC pathogenesis by the immune system.

## 1. Introduction

Colorectal cancer (CRC) is one of the most frequently diagnosed cancers worldwide. According to GLOBOCAN statistics on global cancer, CRC was the third most common cancer and the second leading cause of cancer-related death in 2020 [1]. CRC pathogenesis is caused by three major pathways: chromosomal instability, microsatellite instability, and the CpG island (Cytosine-phosphate-Guanine island; CGI) methylator phenotype (CIMP) [2,3,4]. The CIMP phenotype is found in 30–35% of colorectal adenoma cases [5]. It is characterized by the hypermethylation of CGIs at promoter regions and leads to the inactivation of multiple cancer-related genes, including tumor suppressors [6,7,8,9].

DNA methylation, which is catalyzed by a family of DNA methyltransferases that transfer a methyl group to the 5′-carbon of cytosines in CpG sites [10], plays a critical role in carcinogenesis to control gene expression [11]. In particular, hypomethylation and hypermethylation are related to transcriptional activation and gene silencing, respectively [12,13]. The alteration of DNA methylation has been proposed as a potential biomarker for cancer diagnosis, treatment response prediction, and prognosis, as it occurs in early carcinogenesis [14,15]. Furthermore, some studies have reported that differently methylated and differently expressed genes correlated with prognosis in CRC patients [16,17]. However, the exact role of DNA methylation in gene expression remains unclear [18]. Specifically, the impact of CpG sites in various regions of the gene has not been precisely determined yet [19,20], although many studies have investigated differently methylated CpG sites in CGIs and CGI-surrounding regions, such as the CGI shore (0–2 kb from CGIs), CGI shelf (2–4 kb from CGIs), and open sea (a region with any specific designation) [21,22]. Several studies have also reported that DNA methylation levels in the gene body, particularly in the first exon or first intron, are related to gene expression [23,24,25]. Another recent study detected differentially methylated regions (DMR) between CRC patients and normal groups and annotated their genomic regions, including CGI-related features [26]. In addition, one study found that the effect of DNA methylation on gene expression varies based on the distance between a CpG site and the gene and that the DNA methylation of distant CpG sites can also affect gene expression [27].

This study examined the potential effect of DNA methylation on various genomic areas, including gene components and CGI-related regions, based on their distance from the genes in CRC. To determine the CpG site in which methylation can modulate expression in CRC patients, this study obtained the DNA methylation and transcription profiles from The Cancer Genome Atlas (TCGA) [28] and trained several statistical machine learning models, such as Lasso, Ridge, ElasticNet, and a Bayesian sparse linear mixed model (Bslmm) [29], using DNA methylation values of CpG sites within ±1 Mb of the transcription start sites (TSS). The models have been proposed to solve the problem wherein there are significantly more features than the number of samples, and they have been utilized in various biological fields, e.g., genome-wide association studies [30,31,32,33], transcriptomic profiling [34,35,36], and multi-omics approaches [37,38,39]. The best model with the highest predictive performance was selected, and the effect of CpG sites was analyzed using the coefficients of the best-performing model, taking into account the distance from TSS and the region relative to the gene. In addition, this study validated the effect of CpG sites according to distance from TSS using independent external datasets. Moreover, this study uncovered genes whose expression is affected by DNA methylation and which have a potential association with survival outcomes. Furthermore, this study identified the biological roles of genes aberrantly expressed due to DNA methylation and associated with survival.

## 2. Materials and Methods

### 2.1. TCGA Data Collection and Preprocessing

Gene expression and DNA methylation data of 393 CRC patients were acquired from the TCGA-COAD and TCGA-READ projects using the R package TCGA biolinks version 1.12.0 [40]. Clinical datasets were also collected via the same process, with the exception of four patients who did not have data on survival times. Consequently, the clinical information of 389 CRC patients was used for survival analysis.

TCGA provides mRNA expression values that have already been processed through several steps [41]. FASTQ files were aligned to the GRCh38/hg38 reference genome of GENECODE v22 using STAR [42], and the read counts mapped per gene were quantified using HTSeq [41,43]. We normalized the HTSeq-based raw counts of genes to transcripts per million (TPM) and transformed them into log_2_(TPM + 1). The protein-coding genes whose log_2_(TPM + 1) values were one or more in at least half of the samples were selected in this study. Genes on sex chromosomes or with no probes within ±1 Mb of TSS were excluded from the analysis.

TCGA methylation datasets were based on the Illumina Infinium HumanMethylation450 BeadChip (450k) [44]. DNA methylation levels represent the ratio of methylated probes to the total array intensity, so it has the form of a β value that ranges from 0 to 1. DNA methylation probes containing missing values in >10% of the samples were excluded. Otherwise, missing values were imputed as the median of the remaining available values. Consequently, 12,822 genes and 394,994 DNA methylation probes were selected for this research.

### 2.2. Collection of DNA Methylation Probes for Each Gene

The probe lists and genomic coordinates of 450k were obtained using the IlluminaHumanMethylation450kanno.ilmn12.hg19 0.6.0 package in R. Because the annotation was provided based on hg19, it was converted to hg38 using UCSC liftOver version 1.16.0 in R. To investigate the effect of the DNA methylation level on gene expression regulation, the probes within ±1 Mb of TSS for each gene were chosen as the features of the candidate models. TSS were determined as the start positions of transcripts with the longest exon length for each gene. By collecting probes within ±1 Mb of the TSS of genes, 380,087 DNA methylation probes were finally obtained for the analysis. On one gene, an average of 575 probes was used, with a maximum of 5577 probes. Table S1 shows the total number of probes per gene.

### 2.3. Statistical Machine Learning Model

Multivariate penalized regression methods (Lasso, Ridge, ElasticNet, and Bslmm) were utilized to determine the impact of the DNA methylation of CpG sites on gene expression. Lasso and ElasticNet are sparse models that give more weight to essential features by reducing the coefficients of less effective features to zero. Ridge is a relatively modest shrinkage approach that converges the coefficients very closely to zero. Ridge has the advantage of considering the minor influence of many features compared with Lasso or ElasticNet. These models apply regularization strategies, minimizing the prediction error while avoiding overfitting. These can achieve an appropriate trade-off between bias and variance by adding a penalty term to the cost function. The following equations describe the regularization approaches:

(1) $$Cost=\frac{1}{2N}\;\overset{n}{\underset{i=1}{\sum }}{\left(y_{i}-\beta_{0}-\overset{p}{\underset{j=1}{\sum }}\beta_{j}x_{ij}\right)}^{2}$$

(2) $$L1\;penalty=\sum_{j=1}^{p}\lambda \alpha |\beta_{j}|,\;L2\;penalty=\sum_{j=1}^{p}\lambda \frac{\left(1-\alpha \right)}{2}\beta_{j}{}^{2}$$

(3) $$L\left(\beta ,\beta_{0}\right)=\mathrm{argmin}\left(\beta ,\beta_{0}\right)[\;\mathrm{Cost}\;+L1\;\mathrm{penalty}\;+L2\;\mathrm{penalty}\;]$$

In Equations (1)–(3), n is the number of patients and p is the number of DNA methylation probes in each gene. When y is the gene expression value and x is the DNA methylation level, the shrunken coefficients β for each probe can be obtained by training the model with an adequate shrinkage penalty λ. In Equation (2)**,**  α=1 denotes the Lasso model with the *L*1 penalty, and α=0 denotes ridge regression with the *L*2 penalty. If 0<α <1, ElasticNet, which combines the *L*1 and *L*2 penalties, is used [45]. Collectively, the relative influence of the two penalty terms can be controlled by regulating α.

Bslmm combines the benefits of standard linear mixed models with sparse regression modeling [29,46]. It assumes prior distributions for hyperparameter speciation and infers posterior distributions by efficiently fitting a Markov chain Monte Carlo algorithm. Similar to regularization techniques, Bslmm can bring the coefficients close to zero, excluding a few informative features. More details of Bslmm are described in [29]. Lasso, Ridge, and ElasticNet were implemented via the glmnet package version 4.1.3 in R. The appropriate value of α was searched by altering the α value between 0 and 1 by 0.1. Bslmm was implemented via GEMMA software version 0.98.4.

### 2.4. Performance Evaluation and Model Selection

Five-fold cross-validation was conducted using the cv.glmnet function provided in the glmnet package. The optimal value of λ in Equation (2) was searched automatically through glmnet after cross-validation. The performance of the models was calculated using the metrics in Equations (4)–(7). The mean squared error (MSE), root MSE (RMSE), and mean absolute error (MAE) measure the error of the models, and the R2 score assesses the model’s explanatory power [47]. To find genes whose expression level was affected by DNA methylation, the R2 score was used, because MSE, RMSE, and MAE were scale-dependent measures [48]. When the R2 score is closer to 1, it indicates that the regression model is fitted to the data well.

(4) $$\mathrm{MSE}=\frac{1}{n}\sum^{\;}{\left(y_{real}-y_{pred}\right)}^{2}$$

(5) $$\mathrm{RMSE}=\sqrt{\frac{1}{n}\;\sum^{\;}{\left(y_{real}-y_{pred}\right)}^{2}}$$

(6) $$\mathrm{MAE}=\frac{1}{n}\sum^{\;}\left|y_{real}-y_{pred}\right|$$

(7) $$R2=1-\frac{\sum^{\;}{\left(y_{real}-y_{pred}\right)}^{2}}{\sum^{\;}{\left(y_{real}-Mean\left(y_{real}\right)\right)}^{2}}$$

In all candidate models, the accuracy metrics were calculated in the same way, and the model with a smaller error and higher correlation levels in more genes was selected.

### 2.5. Investigation of the Significance of CpG Sites

The finally selected model, ElasticNet, was retrained using the entire set of 393 patients’ data with the selected α and λ in Equation (2). The coefficient for each feature in regression techniques represents the impact of that feature on the target. Because the range of expression values varies by gene, the absolute values of the coefficients in each gene were scaled via min–max normalization. If the coefficient was equal to zero, it was regarded as a noneffective probe and excluded after the normalization process. Normalized coefficients were used after rounding to one decimal place.

### 2.6. Genomic Annotation for Probes

Using the GENECODE v22 Gene Transfer Format file, the genomic coordinate of each component of the gene body was extracted. The probes located in the gene body were annotated with the component in which they were found. In addition, in order to check the relation to CGI, information about probes located in the target gene’s CGI and the CGI-surrounding regions was retrieved using the IlluminaHumanMethylation450kanno.ilmn12.hg19 0.6.0 package in R. The probes in the CGI-related regions of the target gene were tagged with the corresponding region.

### 2.7. Validation Data Analysis

The CRC cell line gene expression datasets were obtained from the Cancer Cell Line Encyclopedia (CCLE) [49]. DNA methylation datasets for the identical cell lines were downloaded from GSE68379 in the form of β values, which had already been processed by Iorio F. et al. [50]. The cell lines from two different datasets were intersected by name, and, as a result, the gene expression and the DNA methylation data were matched for 31 CRC cell lines (Table S2). The validation data were preprocessed in the same way as in our experiments with TCGA datasets. Consequently, 15,760 genes and 484,843 DNA methylation probes were selected. After collecting probes within ±1 Mb of the TSS of genes, 469,522 DNA methylation probes were used for the validation experiments. The total number of probes per gene can be found in Table S3.

### 2.8. Survival Analysis

For each target gene, the samples were separated into low- and high-expression groups according to the optimal cutoff values determined via the Maxstat R package version 0.7.25, similar to other previous studies that divided groups for survival analysis [51,52,53,54]. A log-rank test was used to examine whether there was a relationship between the expression levels of each gene and survival.

### 2.9. Identification of Signature Genes and Functional Annotation

Methylation-affected and survival-associated (MASA) genes were defined as genes whose expression was affected by DNA methylation and were potentially associated with survival outcomes. In detail, it was considered that gene expression levels were affected by the methylation pattern if the R2 score was >0.3. Additionally, if the *p* value obtained by the log-rank test was ≤0.05 in the survival analysis, the gene expression change had the potential to affect survival. MASA genes were retrieved by intersecting the two groups of genes. To distinguish the potential biological impact of MASA, Gene Ontology (GO) analysis for biological processes (BP) and the Kyoto Encyclopedia of Genes and Genomes (KEGG) pathway enrichment analysis were conducted by DAVID [55]. GO terms and KEGG pathways with a false discovery rate (FDR) of ≤0.05 were extracted as enriched sets.

## 3. Results

### 3.1. Best-Performing Model: ElasticNet

**Figure 1:**
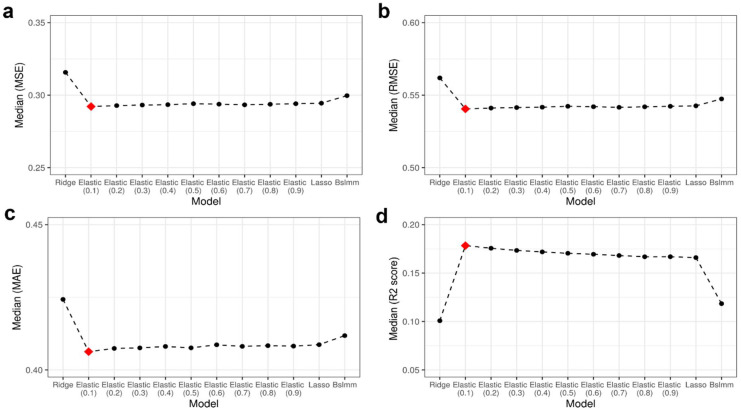
Performance of the candidate models. The numbers in parentheses are the α values of ElasticNet. (**a**) Median MSE for target genes per model; (**b**) median RMSE for target genes per model; (**c**) median MAE for target genes per model; (**d**) median R2 score for target genes per model. The best model is marked with a red diamond in each metric.

To predict gene expression using the DNA methylation levels at CpG sites, four multivariate regression models, namely Lasso, Ridge, ElasticNet, and Bslmm, were applied. The performance of the models was evaluated by comparing the original expression of each gene to the predicted value using five-fold cross-validation. To identify the best model that could accurately predict gene expression for as many genes as possible, the median values in each performance metric, namely MSE, RMSE, MAE, and R2 score, were examined (Figure 1a–d). The R2 score is a value that indicates the explanatory power of the model, and a higher R2 score indicates that the model has better performance. By contrast, MSE, RMSE, and MAE represent the model error; the smaller the value is, the better the model’s performance. ElasticNet, with α = 0.1, had the highest median R2 score (Figure 1d). When α = 0.1, ElasticNet also showed the lowest median error values compared with other methods (Figure 1a–c), although the values were very slightly different among ElasticNet models with different α values. Consequently, ElasticNet with α = 0.1 was selected as the best model, and this model was used in all subsequent analyses.

The number of genes whose R2 scores exceeded the specific values for each model was counted (Table S4). In all models, >12,000 genes had an R2 score of >0. However, as the R2 score increased, the differences between the models became more noticeable. With a higher R2 score, the number of genes decreased rapidly in the case of Bslmm and Ridge. Conversely, in ElasticNet and Lasso, the number of genes decreased slowly, even as the R2 score increased. ElasticNet and Lasso contained >3000 genes with R2 scores of >0.3. Specifically, in ElasticNet with α = 0.1, the R2 scores of 3307 genes were >0.3. This was the largest number of genes among the models, and these genes were regarded as genes whose expression can be influenced by DNA methylation.

### 3.2. Importance of DNA Methylation in a Specific Genomic Region

This study evaluated which CpG sites’ methylation levels affect gene expression using the coefficients of the finally selected ElasticNet model. The normalized coefficients obtained after min–max normalization of the absolute coefficient values of CpG sites within ±1 Mb of TSS were utilized. It was hypothesized that if the coefficient was negative, DNA methylation would negatively affect gene expression. The negative effect means that high DNA methylation levels can cause genes to be underexpressed, whereas low DNA methylation can lead to genes being overexpressed.

**Figure 2:**
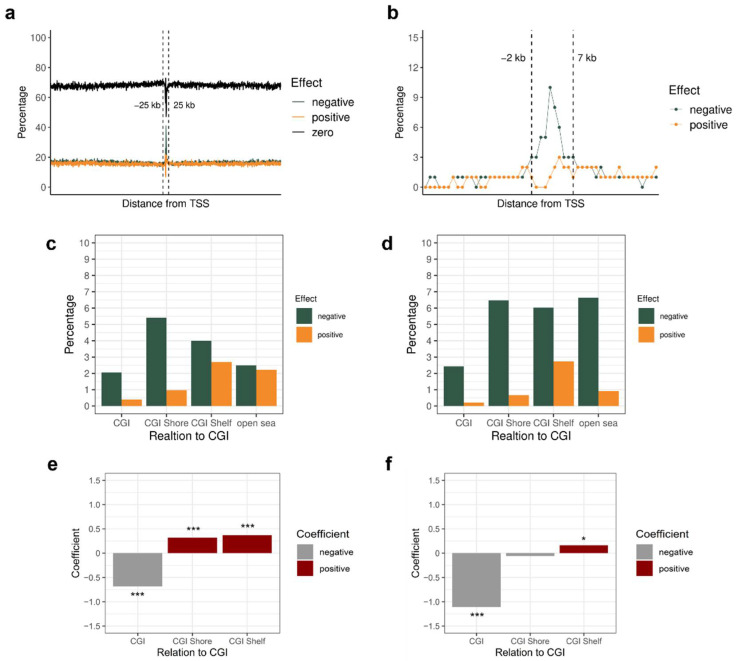
Regulatory effects represented by coefficients of CpG sites. Green, negative effect probes; orange, positive effect probes; black, zero, noneffective. (**a**) Effects of probes according to the distance from TSS. Probes within ±1 Mb of TSS were used. (**b**–**f**) The percentage of probes was represented by dividing the number of probes with absolute normalized coefficients of 1 by the number of probes with absolute coefficients more than zero. (**b**) Effects according to the distance from TSS. Only probes within ±25 kb were plotted. (**c**) Effects according to the relation to CGI within ±1 Mb of TSS. (**d**) Effects according to the relation to CGI within 2 kb upstream to 7 kb downstream of TSS. (**e**,**f**) Comparison of importance among CGI-related regions by logistic regression. The positive coefficient indicates a high probability of important probes in that region (red bar). On the other hand, a negative coefficient means that the probes in that region have less importance (grey bar). The open sea was not represented because the coefficient was not estimated from the logistic regression model. The region where its coefficient was estimated with a considerably low *p* value (*p* < 2.2 × 10^−10^) was marked as ***, and that estimated with *p* < 0.05 was marked as * above the bar. (**e**) Probes within ±1 Mb of TSS were used. (**f**) Probes within 2 kb upstream to 7 kb downstream of TSS were used.

This study investigated in which direction effective CpG sites are located from TSS and how the effects are changed according to the distance from TSS. Moreover, we checked whether the DNA methylation of CpG sites was negatively or positively related to gene expression levels. Most effective CpG sites were located within ±25 kb of TSS (Figure 2a). Therefore, the region of interest was narrowed down within ±25 kb, and the effect of methylation in this area was further analyzed. CpG sites with an absolute normalization coefficient of 1 can be considered important sites for gene expression. To analyze how the percentage of important CpG sites varied with distance, the number of important probes was divided by the number of probes with absolute coefficients more than zero within that distance. Figure 2b shows the percentage of important probes. The percentage was high, especially from 2 kb upstream to 7 kb downstream, and most important CpG sites near TSS had a negative effect rather than a positive one. Moreover, when we restricted our analysis of the regulatory effects only to the genes with high R2 scores (>0.3), we still confirmed that the percentage of the effective probes within ±1 Mb of TSS and the negative effect of the CpG sites from 2 kb upstream to 7 kb downstream of TSS were fairly similar (Figure S1a,b). Because the 2 kb upstream region of TSS was generally contained in a promoter region in previous studies [27,56], the negative effect of DNA methylation at the promoter region on gene expression could be verified again.

Furthermore, we analyzed whether CGIs and their surrounding regions are significant for transcription regulation by DNA methylation. The number of effective probes located in CGI-related regions within ±1 Mb of the TSS of the target gene was investigated. When the percentage of important probes among the number of probes with effects was measured, the percentage of important probes with negative effects was clearly higher than those with positive effects (Figure 2c). The high percentage of important probes with negative effects was also similarly detected when only employing the genes with high R2 scores (>0.3) (Figure S1c).

To identify which region is significantly more important than others, we applied a logistic regression model using the allocation information of the important probes among the CGI-related regions. As a result, the CGI shore and CGI shelf had positive coefficients and low *p* values, which means that there existed significantly more important probes in these regions (*p* < 2.2 × 10^−10^; Figure 2e; Table S7). By restricting the analysis to the genes with high R2 scores, the CGI shore revealed a positive coefficient with a low *p* value, but the estimated coefficient was relatively smaller and the *p* value was considerably higher than the results obtained by all genes (*p* < 2.2 × 10^−5^; Figure S1e; Table S7).

Furthermore, to identify the effect of CGI-related regions within 2 kb upstream to 7 kb downstream of TSS, the percentage of important probes among the number of effective probes was reinvestigated (Figure 2d). All CGI-related regions had a high percentage of important probes with a negative effect. This tendency was also clearly discovered in the results of TCGA datasets with genes with high R2 scores (Figure S1d). When utilizing a logistic regression model, the CGI shelf was more important than other CGI-related regions with a positive coefficient and a low *p* value (*p* < 0.05; Figure 2f; Table S8). However, the coefficient and *p* value were not so significant compared to the results from CGI-related regions within ±1 Mb of the TSS of the target gene. Moreover, when analyzing genes with high R2 scores, important CGI-related regions with a positive coefficient were not detected (Figure S1f; Table S8).

Interestingly, regardless of the R2 scores of genes, more than 80% of the important probes with a negative impact within ±1 Mb were retained in the CGI, shore, and shelf within 2 kb upstream to 7 kb downstream of TSS (Tables S5 and S6). Therefore, this study validated that DNA methylation within 2 kb upstream to 7 kb downstream of TSS is important for expression regulation, and the DNA methylation-medicated gene regulation has a relatively weak relationship with CGIs in this area.

For further validation, we carried out the same experiments using CCLE CRC cell line datasets. The percentage of effective probes was increased near TSS, similarly to TCGA datasets (Figure S2a). Moreover, the CpG sites with negative effects were mainly observed from 2 kb upstream to 7 kb downstream (Figure S2b). In the CGI-related regions, excluding the open sea, there were more probes with negative effects than positive ones (Figure S2c). When we restricted the analyzed region to 2 kb upstream to 7 kb downstream, it was confirmed that the probes with negative effects were more frequently located in all CGI-related areas, including the open sea (Figure S2d). It was noted that more than 90% of the important probes with a negative impact were retained in the CGI, shore, and shelf, within 2 kb upstream to 7 kb downstream of TSS (Tables S9 and S10). There were no important CGI-related regions obtained by the logistic regression analysis both within ±1 Mb (Figure S2e; Table S11) and within 2 kb upstream to 7 kb downstream of TSS (Figure S2f; Table S12).

**Figure 3:**
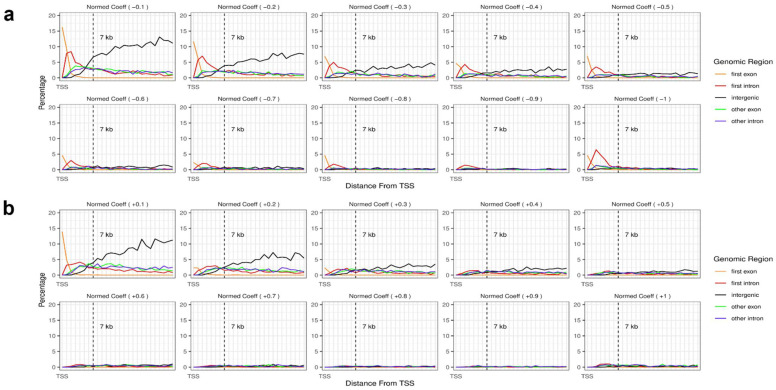
Regulatory effects represented by coefficients of CpG sites within genes. Effects of the probes within genes with (**a**) negative coefficients and (**b**) positive coefficients according to the distance from the TSS. The *x*-axis is the relative distance from TSS, and the leftmost point of the *x*-axis is TSS. The *y*-axis is the percentage of probes represented by dividing the number of probes with absolute normalized coefficients of 1 by the number of probes with absolute coefficients more than zero. The colors of the lines denote the genomic regions. Orange, first exon; red, first intron; black, intergenic; green, other exon; blue, other intron. Other exon/intron refers to all exons/introns in the transcript except the first exon/intron. Only downstream regions are plotted.

This study also investigated genomic regions at ~7 kb downstream and the regional effects on gene expression. The probes with normalized coefficient values of −1 were more likely to be found in the first intron region and first exon (Figure 3a). Conversely, probes with highly positive effects, denoted as normalized coefficients of 1, did not specify any representative regions (Figure 3b). Interestingly, a considerable percentage of the probes with low normalized coefficients were found in intergenic regions, regardless of whether the effects were negative or positive. Moreover, the percentage of probes with low coefficients at intergenic regions moderately increased with the distance from TSS (Figure 3a,b). Furthermore, in both the experiments only using genes with high R2 scores and the validation experiments using CCLE datasets, the first intron and the first exon were also found to be important regions, where DNA methylation occurring in these regions negatively affects gene expression (Figures S3 and S4).

### 3.3. Prediction with Probes between 2 kb Upstream and 7 kb Downstream of TSS

The DNA methylation of CpG sites in the region from 2 kb upstream to 7 kb downstream of TSS has an important role in gene regulation. This study assessed the model’s prediction performance using only the probes within the corresponding regions.

**Figure 4:**
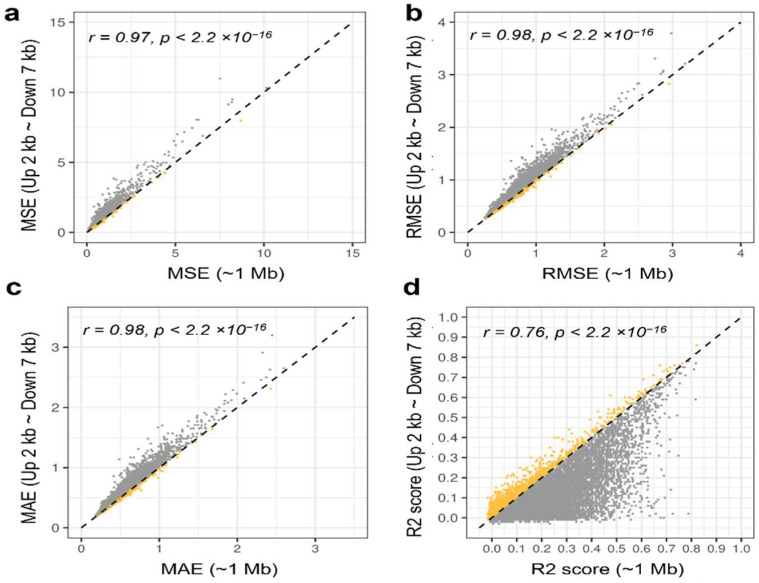
Performance comparison of ElasticNet depending on probe distance. Gene expression prediction was performed with ElasticNet using probes within ±1 Mb of TSS and ElasticNet using probes located in the region of 2 kb upstream to 7 kb downstream. Each model’s performance was measured and compared in terms of (**a**) MSE, (**b**) RMSE, (**c**) MAE, and (**d**) R2 score. Genes with better performance using probes within 2 kb upstream to 7 kb downstream are highlighted in yellow. The letter r denotes Pearson’s correlation coefficient, and *p* is the *p* value for Pearson’s correlation.

The prediction results using only probes within 2 kb upstream to 7 kb downstream of TSS showed a strong correlation with the results using all probes within ±1 Mb of TSS (Figure 4a–d). Additionally, some genes showed slightly better performance when only the probes of the regions (2 kb upstream to 7 kb downstream) were used. These findings suggested that the DNA methylation levels in 2 kb upstream to 7 kb downstream regions are crucial for regulating gene expression. However, the effect of DNA methylation on a wide range of regions around TSS cannot be ignored.

### 3.4. MASA Genes

**Figure 5:**
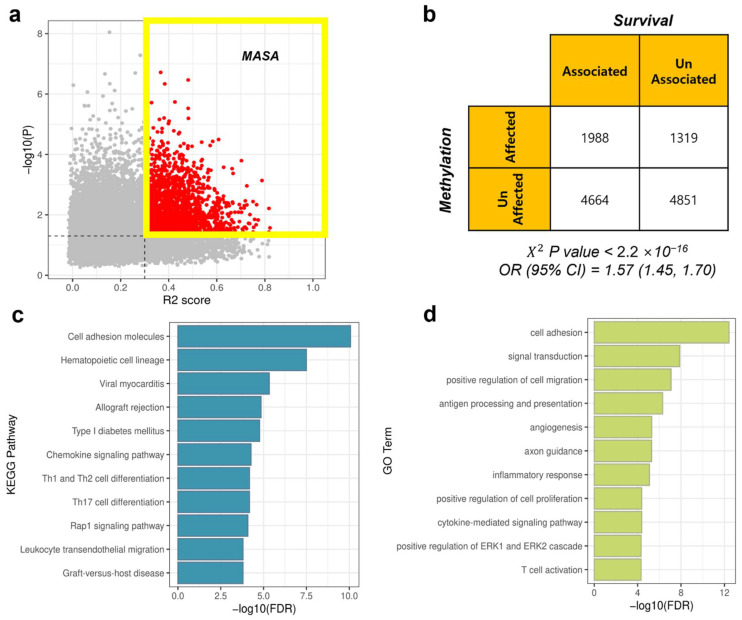
Analysis of MASA genes. (**a**) Comprehensive view of each gene. Red dots are MASA genes with R2 score > 0.3 and *p* ≤ 0.05. (**b**) Number of genes classified corresponding to each specified characteristic. (**c**) KEGG pathway enrichment analysis using MASA. (**d**) GO BP enrichment analysis using MASA.

To conduct survival analysis, patients were divided into two groups according to gene expression levels (see Methods). Signature genes whose expression is affected by the DNA methylation level and has the potential to affect survival rates were defined as MASA genes (R2 score > 0.3 and *p* ≤ 0.05, log-rank test; Figure 5a). Whether DNA methylation-affected genes are related to the survival rate was assessed using Pearson’s χ^2^ test. The *p* value was very low (*p* < 2.2 × 10^−16^; Figure 5b). Genes whose expression is affected by DNA methylation were statistically linked to patient survival. Additionally, the odds ratio (OR) was 1.57, indicating that DNA methylation-affected genes were 1.57 times more relevant to survival than non-methylation-affected genes. The 95% confidence interval of the OR was 1.45–1.70.

Finally, the KEGG pathway and GO enrichment analysis for BP were conducted to investigate the biological roles of MASA genes and explore how they are related to survival. MASA genes were mainly enriched in immune-related pathways (Figure 5c). The two most significantly enriched pathways were cell adhesion molecules and hematopoietic cell lineage pathways, and both were also related to the immune system. Cell adhesion molecules play a crucial role in all aspects of inflammation, such as mediating the migration of immune cells near malignant tumor cells or regulating the interaction between immune cells or between immune cells and target cells [57]. Moreover, hematopoietic stem cells (HSC) are a raw form of blood cells that can differentiate into any type of immune cell lineage: either lymphoid lineages, such as T and B cells, or myeloid lineages, such as macrophages or granulocytes [58]. Therefore, hematopoietic cell lineage pathways are involved in all differentiation processes of HSC to immune cell lineages. Likewise, the most enriched GO BP terms were also related to the immune response (Figure 5d). Notably, MASA genes were enriched in the angiogenesis process (Figure 5d), which plays an important role in cancer cell growth and proliferation [59,60].

## 4. Discussion

CRC is one of the cancers most commonly diagnosed worldwide and has a high mortality rate [61]. CRC pathogenesis can be influenced by various abnormal genetic or epigenetic modifications [62,63,64]. DNA methylation is one of the most important epigenetic features, in which abnormal changes can lead to CRC development by disrupting transcription regulation [65]. Using multivariate regression models, this study investigated how DNA methylation affects gene expression. To determine the model that can explain the relationship between DNA methylation and gene expression, the models’ performance was evaluated. Consequently, ElasticNet outperformed all others in terms of the lowest median error and the highest median R2 score for target genes. The weights of most sites were offset by zero in ElasticNet. Therefore, it was concluded that DNA methylation changes at all CpG sites do not affect gene expression levels uniformly and that specific, important CpG sites can more strongly regulate transcriptional events.

To identify CpG sites with a strong influence, the coefficients for each probe were analyzed. Therefore, DNA methylation within 2 kb upstream to 7 kb downstream of TSS was proven more essential in regulating gene expression than other CpG sites. Specifically, DNA methylation in these regions can affect gene expression negatively. Because the promoter region is generally located upstream of the TSS [66,67], it was reconfirmed that DNA methylation at the promoter regions inhibits gene expression. Furthermore, by investigating the distribution of probes in CGI-related regions, we investigated the regulatory effects exerted by the CGI-related regions, because some previous studies reported that DNA methylation in the CGI shore and CGI shelf was important [68,69,70,71,72]. Our study also suggested that the CGI was less effective in gene regulation and the CGI shore and CGI shelf regions were relatively more important. However, based on the results obtained using genes with high R2 scores, the regulatory effect would be limited, especially at the regions near the TSS.

Interestingly, the relevant CGI-related regions with clearly low *p* values were not found in the 2 kb upstream to 7 kb downstream region. The *p* values of the regions with positive effects were also more than 0.01 in this area. This result would imply that the relation to CGI is less significant because important probes with negative effects are mainly concentrated in the 2 kb upstream to 7 kb downstream region. Moreover, in the CCLE datasets utilized for the validation experiments, no important CGI-related regions were detected. These results may be due to the characteristics of cell lines being different from tissue samples from cancer patients, or the limitation of the low number of validation samples (*n* = 31). However, given that most of the important probes with negative effects in CGI-related regions, in the CCLE datasets, were located in the region 2 kb upstream to 7 kb downstream of TSS, the result might make sense. To better evaluate the importance of the CGI-related region, further computational and biological experimental studies using more datasets would need to be carried out.

Additional analyses were also conducted to explore which gene components can support the negative effect of the 7 kb downstream region. Consequently, DNA methylation occurring in the first exon and the first intron located in the region 7 kb downstream of TSS could be the key component that can negatively affect transcription regulation. This means that hypermethylation or hypomethylation in the first exon and first intron can cause gene silencing or overexpression, respectively, and may eventually lead to CRC pathogenesis.

To verify which DNA methylation pattern within 2 kb upstream to 7 kb downstream of TSS can sufficiently explain the expression level, ElasticNet was reconstructed only using the probes in this region. When their performance was compared, the Pearson’s correlation coefficients were very high, reflecting that DNA methylation in the region within 2 kb upstream to 7 kb downstream of TSS can effectively explain gene expression levels. However, many genes showed better performance when utilizing probes within ±1 Mb. It was suggested that the DNA methylation pattern of many CpG sites, albeit at low levels, has some effects on the regulation of gene expression, although the DNA methylation status of specific CpG sites plays a critical role in gene regulation.

Furthermore, this study searched for genes whose expression changes were associated with survival rates. Genes statistically associated with survival were intersected with DNA methylation-affected genes. Finally, 1988 genes whose transcriptional changes are affected by DNA methylation levels and whose expression can be related to survival were identified. The identified signature genes were named MASA, and biological functions enriched in MASA were explored. Obviously, genes were mainly related to immunity. Many studies have already recognized immune systems as a hallmark of cancer [73,74]. The complex interaction between cancer and the immune system can enhance or suppress cancer development and progression [75]. When immune systems function normally, tumor-infiltrating immune cells can eliminate malignant cells and are associated with prognosis [76]. GO analysis also revealed immune-related responses, such as cell adhesion, signal transduction, antigen processing and presentation, and the inflammatory response [57,77,78]. Additionally, MASA is enriched in the angiogenesis process, which is one of the most essential characteristics of cancer [59]. Because cancer cells require nutrients and oxygen for survival, malignant cells are located near blood vessels to obtain essential elements [79].

Due to the importance of DNA methylation in CRC pathogenesis, it is crucial to thoroughly analyze the connection between DNA methylation and gene expression. In addition, one study, by Kerachian et al., identified CRC detection biomarkers based on the DNA methylation status [26]. Our study not only analyzed the relationships between DNA methylation and gene expression patterns occurring in the CGI-related regions, gene body, and promoter, but also revealed that the first exon and first intron were the most important regions in the gene body. Furthermore, the negative effects on gene expression regulation by DNA methylation within 2 kb upstream to 7 kb downstream of TSS was confirmed. Finally, our study identified GO and KEGG pathways in which methylation-affected and survival-associated genes are enriched. Thus, these results could help in the identification of novel biomarkers for the diagnosis and prognosis of CRC patients.

## 5. Conclusions

In summary, this study discovered that DNA methylation within 2 kb upstream to 7 kb downstream regions of TSS can induce aberrant expression, and that genes affected by DNA methylation can be further associated with survival. Because these genes are related to the immune or angiogenesis process, the misfunction of these genes might negatively affect the prognosis of CRC patients.

## Data Availability

All the datasets used in this study are publicly available on the TCGA data portal. Available online: https://portal.gdc.cancer.gov/ (accessed on 5 March 2022), CCLE. Available online: https://sites.broadinstitute.org/ccle/ (accessed on 7 September 2022), and Gene Expression Omnibus (GEO) database (GEO accession: GSE68379).

## Supplementary Materials

The following supporting information can be downloaded at: https://www.mdpi.com/article/10.3390/biology11101388/s1, Figure S1: Regulatory effects represented by coefficients of CpG sites for genes with high R2 scores. That estimated with a low *p* value (*p* < 2.2 × 10−5) was marked as **. The format is the same as in Figure 2; Figure S2: Regulatory effects represented by coefficients of CpG sites in CCLE validation datasets. The format is the same as in Figure 2; Figure S3: Regulatory effects represented by coefficients of CpG sites within genes with high R2 scores. The format is the same as in Figure 3; Figure S4: Regulatory effects represented by coefficients of CpG sites within genes used in the CCLE validation datasets. The format is the same as in Figure 3; Table S1: The number of DNA methylation probes for target genes (*n* = 12,822) used for gene expression prediction; Table S2: Matched information of each CRC cell line from different validation datasets (CCLE + GSE68379); Table S3: The number of DNA methylation probes for target genes (*n* = 15,760) used for validation; Table S4: The number of genes whose R2 scores exceed the specific values for candidate models; Table S5: The number of probes in CGIs and surrounding regions according to effects (probes within ±1 Mb of TSS); Table S6: The number of probes in CGIs and surrounding regions according to effects (probes within 2 kb upstream ~ 7 kb downstream of TSS); Table S7: The results of binary logistic regression model. The number of probes was calculated according to their importance. Only effective probes within ±1 Mb of TSS were counted; Table S8: The results of binary logistic regression model using effective probes within 2 kb upstream ~ 7 kb downstream of TSS; Table S9: The number of probes used in the CCLE validation datasets according to CGI-related regions and probe’s effects (probes within ±1 Mb of TSS); Table S10: The number of probes used in the CCLE validation datasets according to CGI-related regions and probe’s effects (probes within 2 kb upstream ~ 7 kb downstream of TSS); Table S11: The results of binary logistic regression model using the CCLE validation datasets. Only effective probes within ±1 Mb of TSS were used; Table S12: The results of binary logistic regression model using the CCLE validation datasets. Only effective probes within 2 kb upstream ~ 7 kb downstream of TSS were used.
