# Supplementary figures and images for: Genomic Effect of DNA Methylation on Gene Expression in Colorectal Cancer
Source: Biology (Basel). 2022 Sep 23;11(10):1388. doi: 10.3390/biology11101388 (PMC9598958; doi:10.3390/biology11101388)

**a**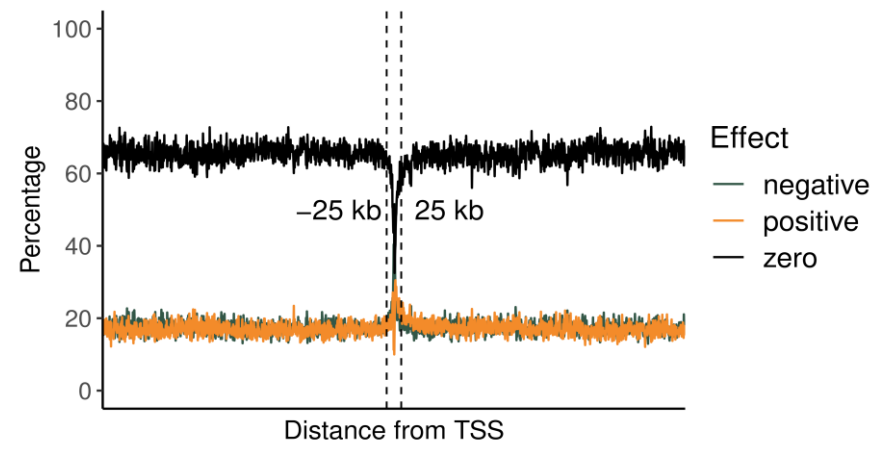**b**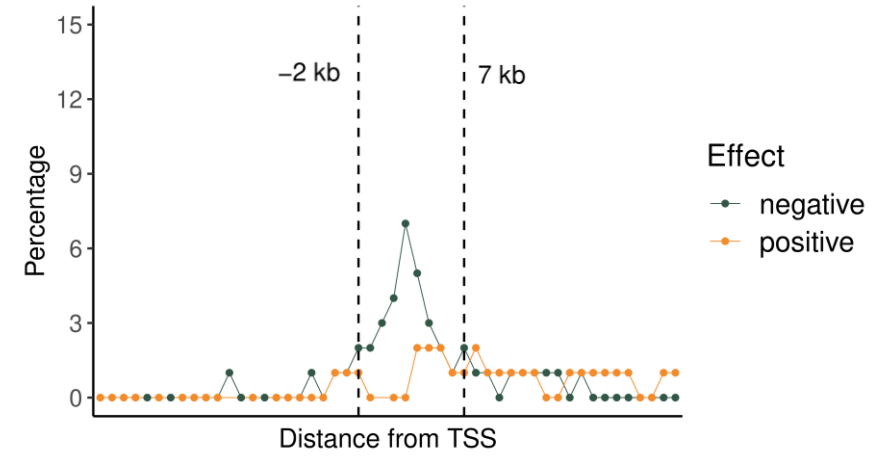**c**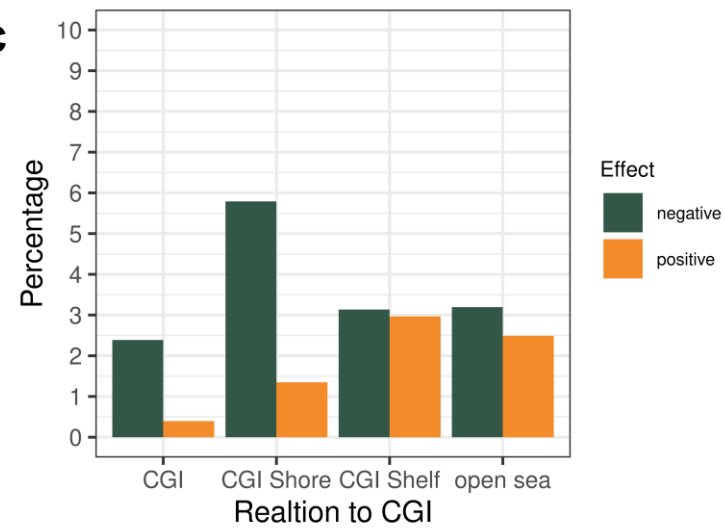**d**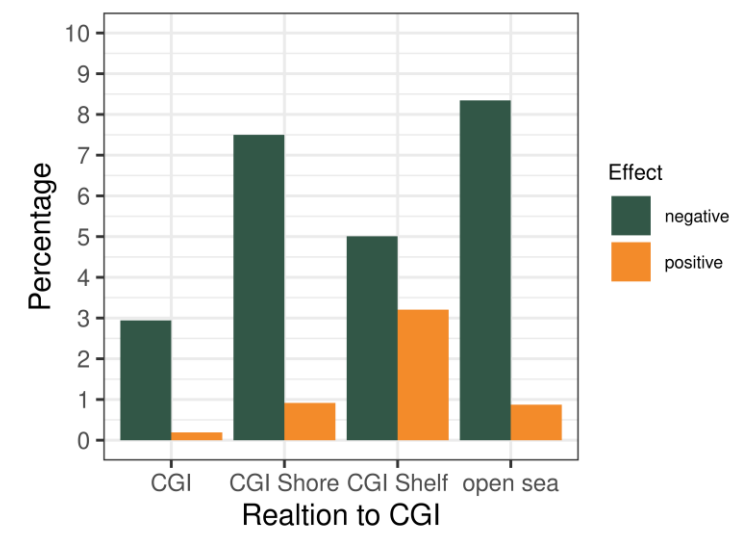**e**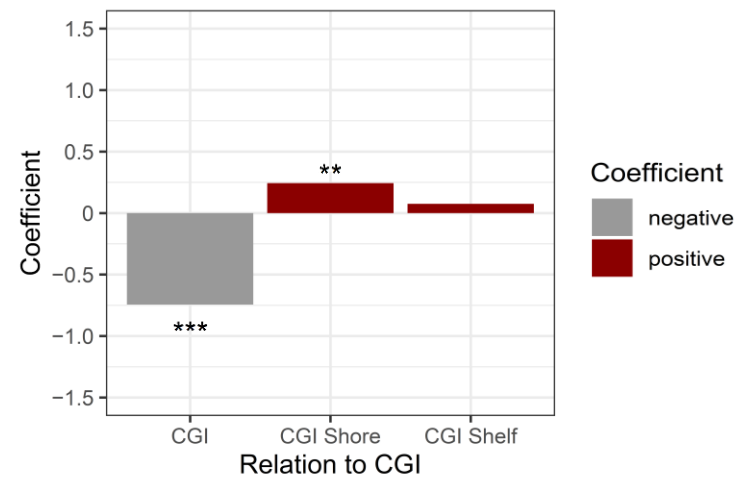**f**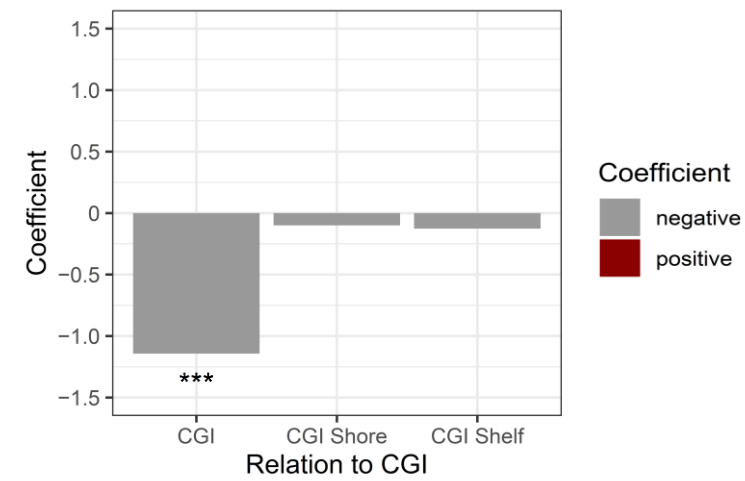

Supplement: Supplementary file 1 [file biology-11-01388-s001.zip › biology-1901940-supplementary (table+figure)/Figure S1.pdf]

**a**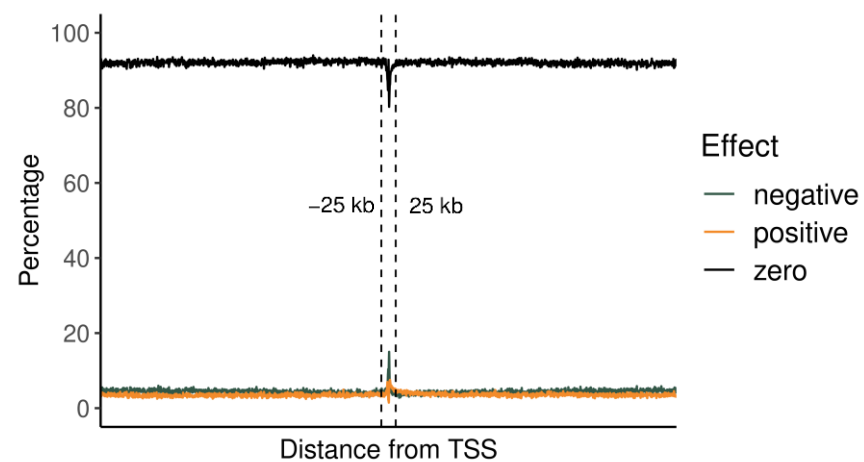**b**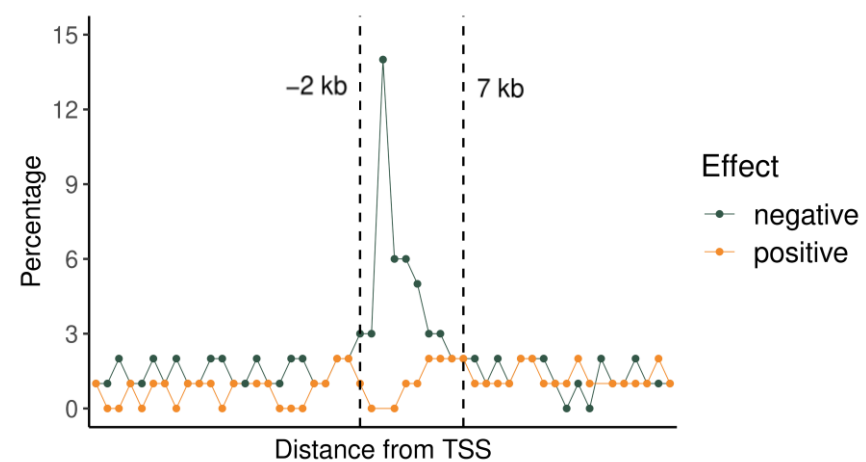**c**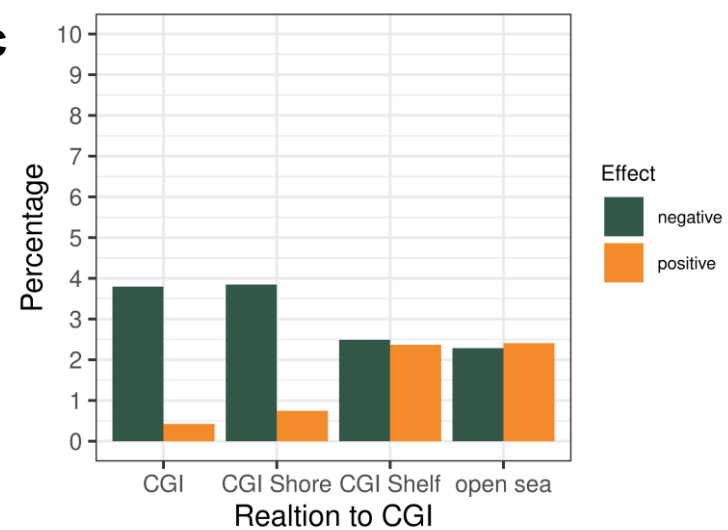**d**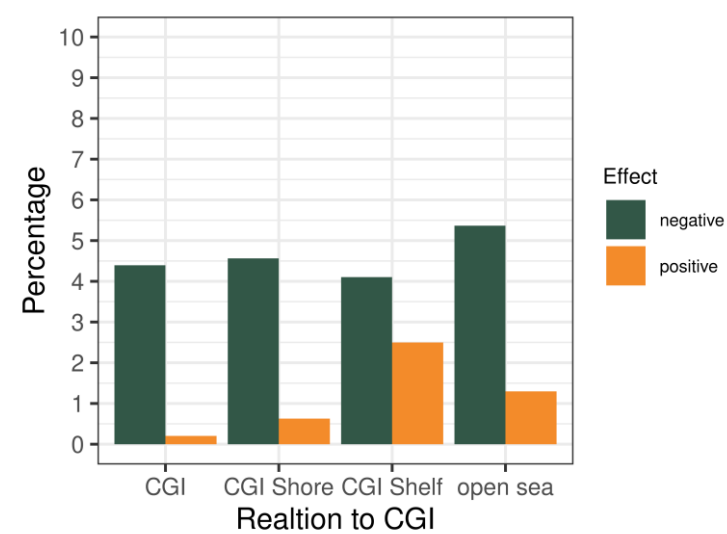**e**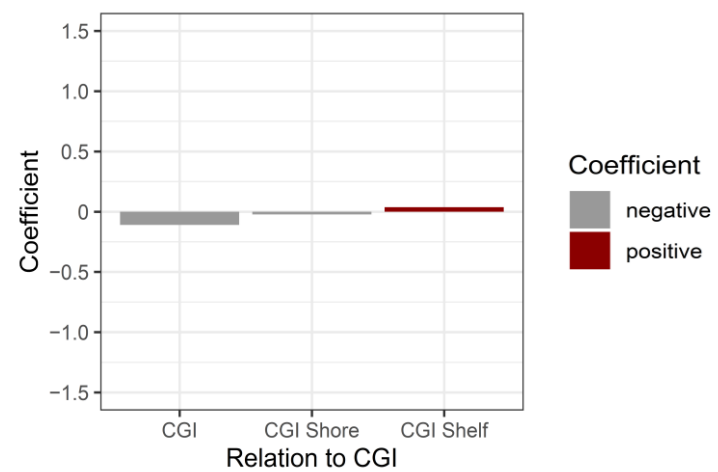**f**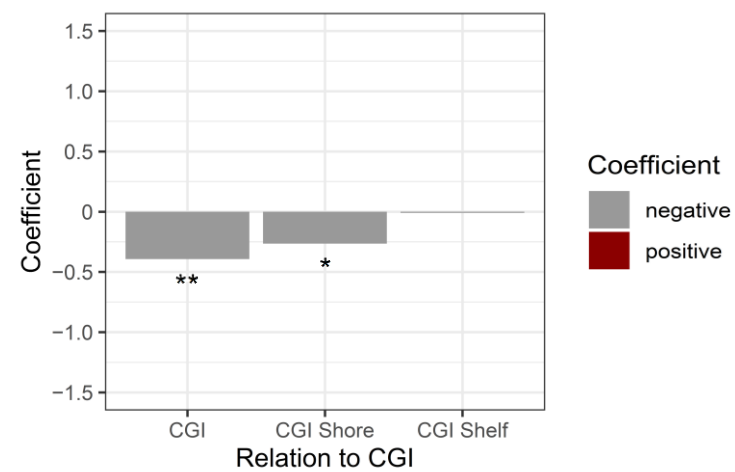

Supplement: Supplementary file 1 [file biology-11-01388-s001.zip › biology-1901940-supplementary (table+figure)/Figure S2.pdf]

**a**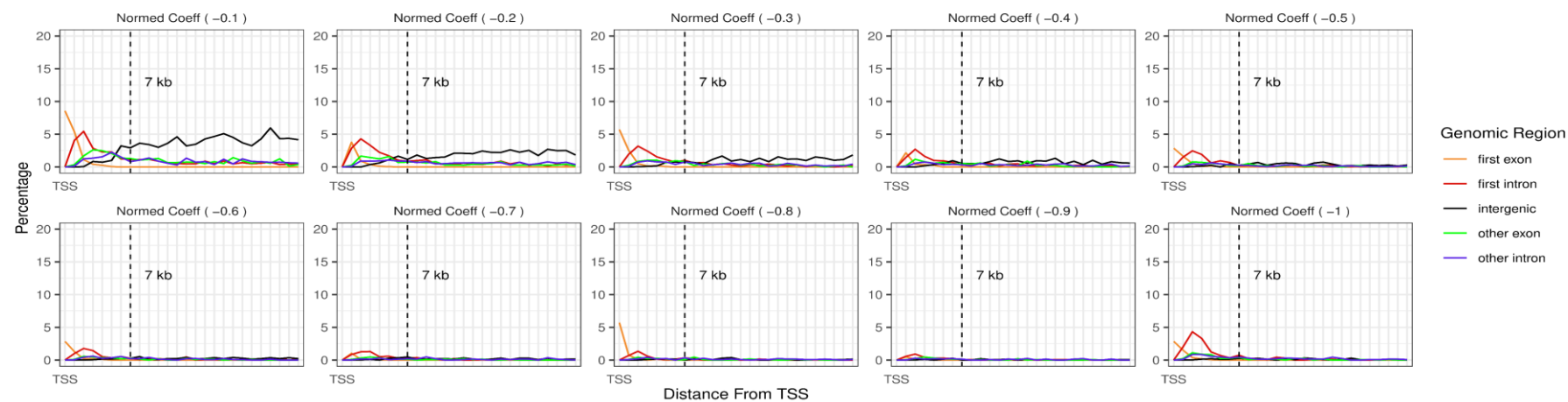**b**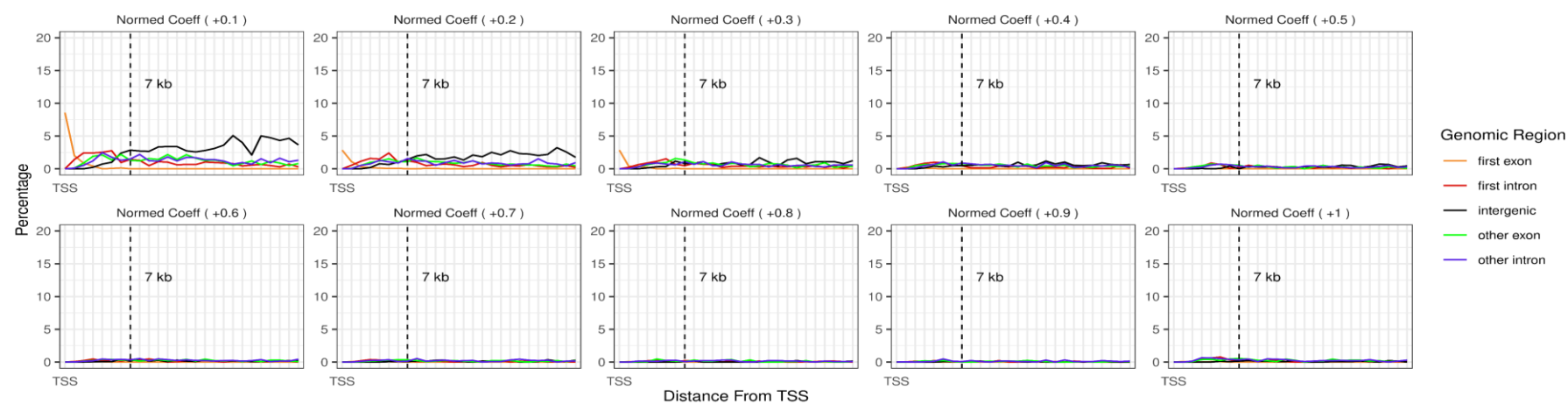

Supplement: Supplementary file 1 [file biology-11-01388-s001.zip › biology-1901940-supplementary (table+figure)/Figure S3.pdf]

**a**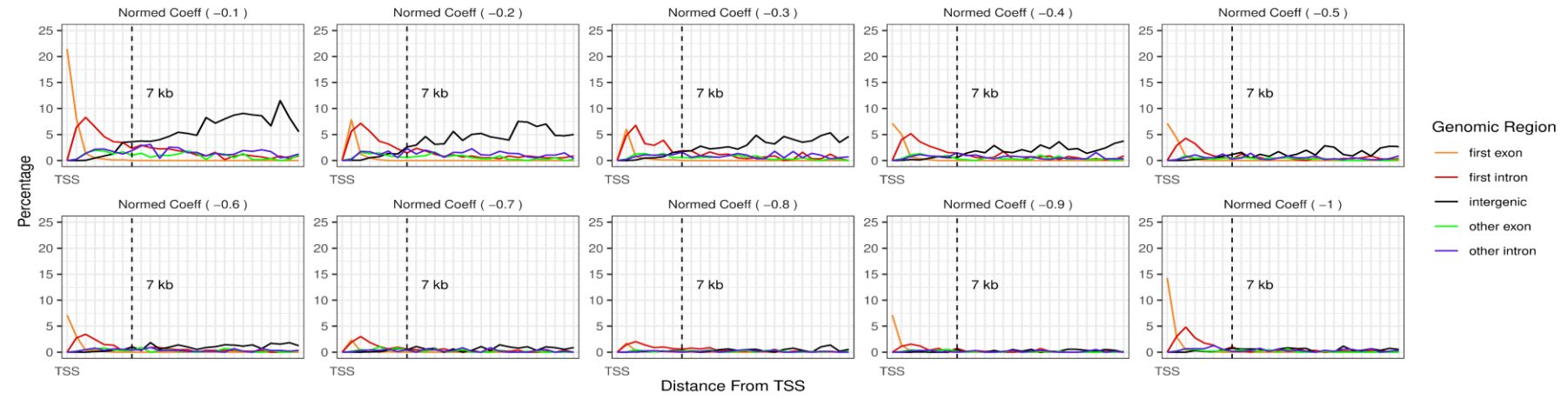**b**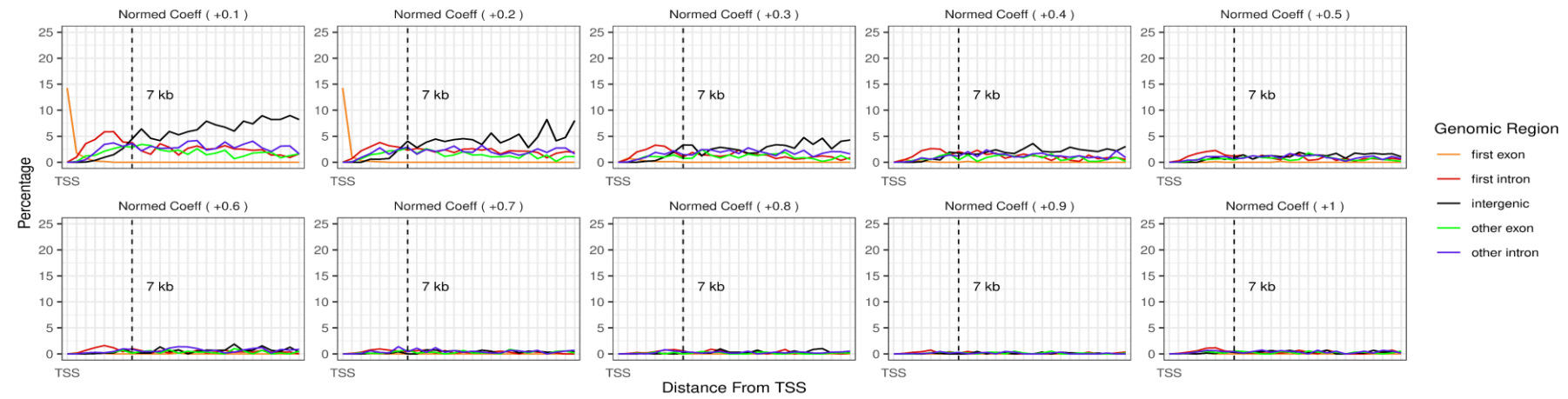

Supplement: Supplementary file 1 [file biology-11-01388-s001.zip › biology-1901940-supplementary (table+figure)/Figure S4.pdf]
